# Unilateral premature osteoarthritis of the hip with excessive anteversion of the femoral neck developing in the early second decade: two surgical cases

**DOI:** 10.1186/s12891-021-04386-3

**Published:** 2021-06-05

**Authors:** Takahiro Nishimura, Hideaki Watanabe, Naoya Taki, Saki Onuma, Ichiro Kikkawa

**Affiliations:** 1 Department of Orthopaedics Surgery, Jichi Medical University, 3311-1 Yakushiji, Tochigi Prefecture Shimotsuke, Japan; 2 Department of Paediatric Orthopaedics and Orthopaedic Surgery, Jichi Children’s Medical Center Tochigi, Tochigi Prefecture Shimotsuke, Japan

**Keywords:** Premature osteoarthritis of the hip, anteversion of the femoral neck, early second decade, proximal femoral flexional derotation varus osteotomy, triple pelvic osteotomy

## Abstract

**Background:**

Osteoarthritis (OA) of the hip rarely develops in the early second decade. As the incidence of this disease is low, no treatment method has been established. We report two patients with unilateral OA in their early teens in whom the anteversion angle of the femoral neck on the affected side was greater than that on the unaffected side.

**Case presentation:**

Case 1 was an 11-year-old girl with left coxalgia and limited range of motion. There was no history of femoroacetabular impingement (FAI) or developmental dysplasia of the hip (DDH). Plain X-rays revealed the disappearance of the Y cartilage, joint space narrowing of the left hip, and acetabular/femoral head osteosclerosis. In CT images, the anteversion angle of the femoral neck (lt/rt) was 45/35 degrees. As osteoarthritis was severe, proximal femoral flexional derotational varus osteotomy (PFFDVO) and triple pelvic osteotomy (TPO) were performed.

Case 2 was a 13-year-old girl with left coxalgia and limited range of motion. There was no history of FAI or DDH. Plain X-ray revealed irregularity of the left anterolateral femoral head, and a subcartilaginous cyst. In CT images, the anteversion angle of the femoral neck (lt/rt) was 30/20 degrees. As osteoarthritis was severe, PFFDVO was performed. In addition, we resected bone spurs on the femoral head because flexion was limited owing to the presence of osteophytes. In both patients, coxalgia and claudication/gait disorder resolved postoperatively, and joint space narrowing and osteosclerosis improved. However, in Case 1, there was a 3-cm difference in the leg length, and in Case 2, range-of-motion limits remained.

**Conclusions:**

We present the findings in two patients with unilateral OA in their early second decade in whom the femoral anteversion angle on the affected side was greater than that on the unaffected side. PFFDVO + TPO was performed in Case 1, and PFFDVO + bone spur resection on the femoral head was performed in Case 2. Coxalgia resolved, and plain X-ray demonstrated improvements in OA; however, a difference in the leg length and range-of-motion limits remained.

## Background

Osteoarthritis (OA) of the hip rarely develops in the early second decade, but hip disorder or pain may remain throughout life unless sufficient treatment is performed. As the incidence of this disease is low, no treatment method has been established. Etiological factors for OA in the early teens include sports-related femoroacetabular impingement (FAI) [1–3] and developmental dysplasia of the hip (DDH) [4, 5]. However, the details remain to be clarified. The anteversion angle of the femoral neck is large in infancy and childhood, and decreases with growth, as reported for the femoral neck shaft angle [6]. A previous study found no reduction in this angle in many patients with DDH [7], but whether the future development of OA depends on this angle in DDH-free patients remains unclear [8].

We report two patients with unilateral OA in the early second decade, with no history of FAI or DDH in whom the anteversion angle of the femoral neck on the affected side was greater than that on the unaffected side.

## Case presentation

### Case 1

The patient was an 11-year-old girl with a 1-year history of left coxalgia that had gradually worsened over time and made walking difficult. She was brought to our hospital for evaluation. There was no history of coxalgia, claudication, trauma, DDH, or generalized joint laxity, and she had not actively participated in sports.

The range of motion in her hip joints at the initial consultation (lt/rt) was 120/120 degrees of flexion, 20/40 degrees of abduction, 30/40 degrees of internal rotation, 30/40 degrees of external rotation, and 10/10 degrees of adduction. On abduction, internal rotation, and external rotation, the range of motion was limited. The flexion, abduction, and internal rotation (FADIR) test demonstrated negative reactions in bilateral hips.

**Fig. 1:**
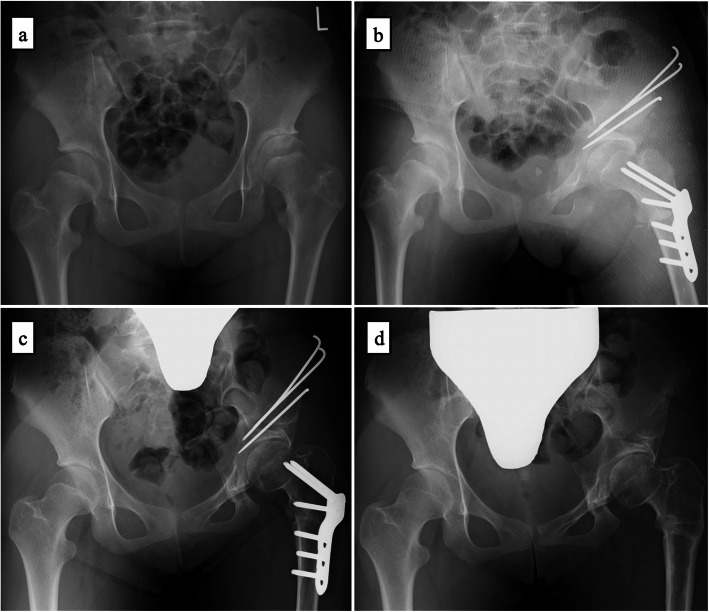
Radiography AP view (a: preoperation, b: postoperation, c: 1 year after surgery, d: 2 years after surgery). a: Joint space narrowing of the left hip and acetabular/femoral head osteosclerosis are seen. Lateral center-edge, Tonnis, and Sharp’s angles (lt/rt) were 25/25, 10/10, and 45/45 degrees, respectively. There were no signs of femoroacetabular impingement. b: Immediately after surgery. The surgical side was fixed with a hip spica cast. c: 1 year after surgery. Improvements in joint space narrowing and acetabular/femoral head osteosclerosis are seen. d: 2 years after surgery. Implant removal

Plain X-ray revealed the disappearance of the Y cartilage, joint space narrowing in the left hip, and acetabular/femoral head osteosclerosis. The lateral center-edge angle (LCEA) (lt/rt), Tönnis angle, and Sharp’s angle were 25/25, 10/10, and 45/45 degrees, respectively. The head–neck offset ratio was 0.25. The alpha angle was 39 degrees. There was no disruption of Shenton’s line, and no acetabular retroversion, cross-over sign, pistol grip deformity, or herniation pit on either side (Fig. 1a).

**Fig. 2:**
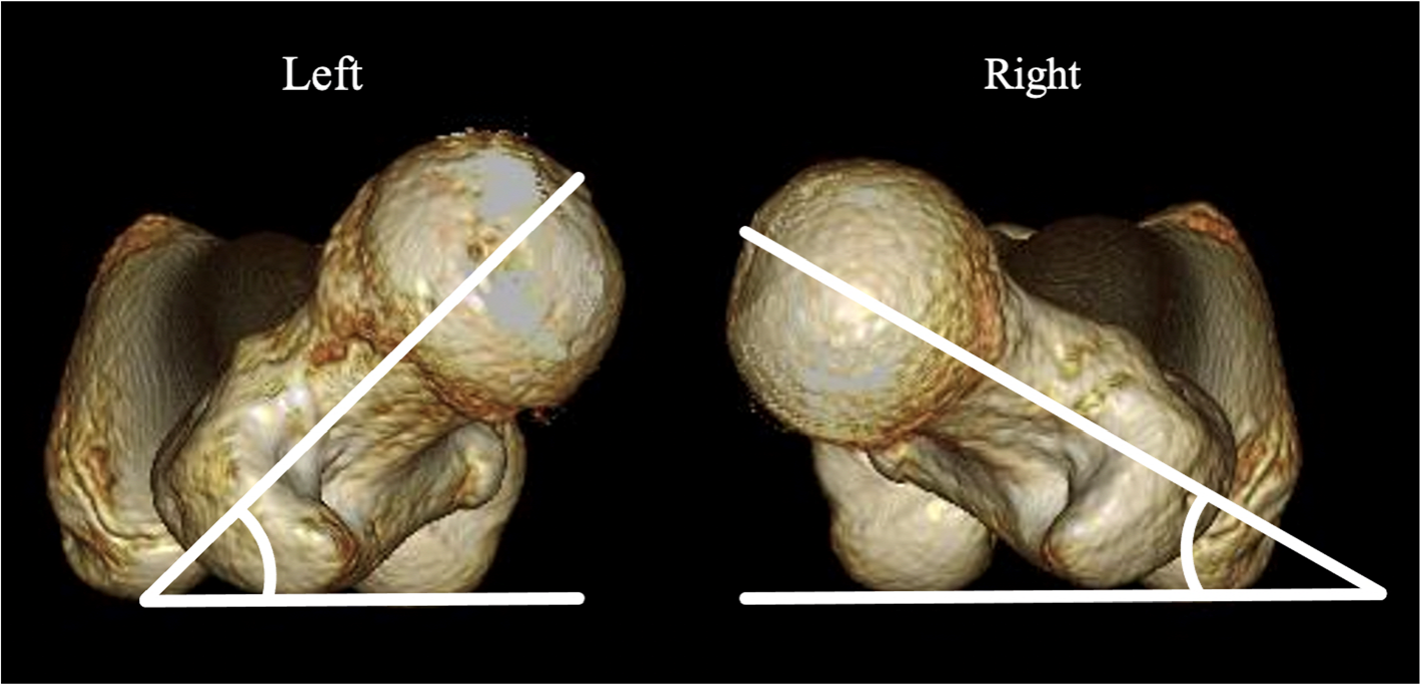
Plain 3D-CT. The anteversion angle of the femoral necks (left/right) was 45/35 degrees. The angle was measured at a point where a tangent on the posterior surface of the medial–lateral femoral condyle overlapped the posterior surface of the greater trochanter when viewing the femur from the cephalic side in the caudal direction

**Fig. 3:**
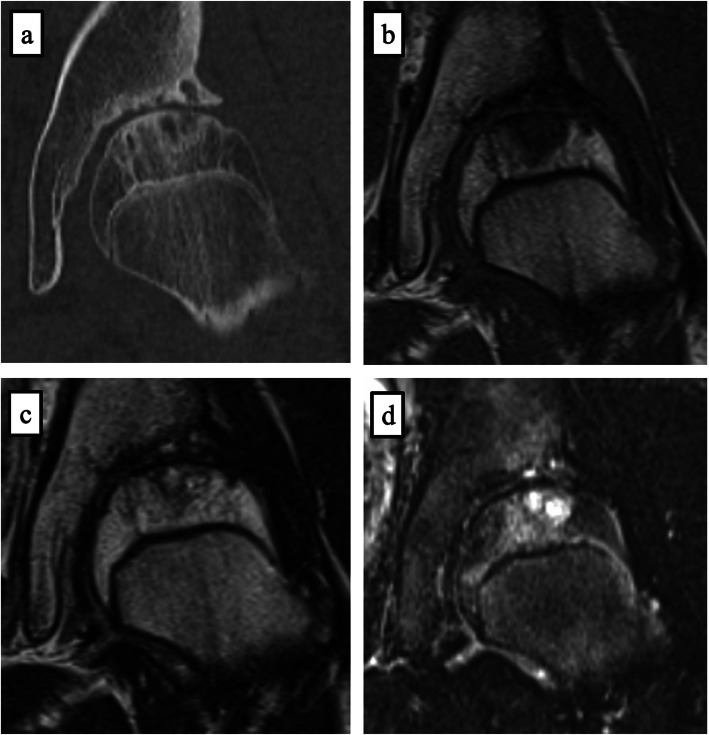
Plane CT and MRI (a: Coronal view of plane CT, b: T1 weighted image, c: T2 weighted image, d: Short TI inversion Recovery). Subcartilaginous cyst in the femoral head and edema were observed

Computed tomography (CT) revealed joint space narrowing of the left hip, and osteosclerosis of the lateral superior anterior acetabulum and anterolateral femoral head, as observed on plain X-ray. In addition, subcartilaginous cysts in the acetabulum and femoral head were detected. The anterior center edge angle (ACEA) for the evaluation of acetabular coverage (lt/rt) was 50/50. The femoral neck shaft angle (lt/rt) was 130/130 degrees, and the angle of anteversion of the femoral neck (lt/rt) was 45/35 degrees [9] (Fig. 2). A subcartilaginous cyst in the left femoral head was observed on magnetic resonance imaging (MRI), as noted on CT. However, there was no necrosis of the femoral head (Fig. 3). There were no abnormalities in the blood test.

A diagnosis of unilateral premature OA of the left hip with excessive anteversion of the femoral neck was made. As osteoarthritis was severe, surgery was selected. To improve hip conformity, reduce anteversion of the femoral neck, and alter the weighted surfaces of the acetabulum and femoral head, proximal femoral flexional derotation varus osteotomy (PFFDVO) (flexion: 20 degrees, detorsion: 20 degrees, inversion: 20 degrees) and triple pelvic osteotomy (TPO) [10] were performed for the following reasons. Firstly, hip conformity on extension/internal rotation/abduction was favorable. Secondary, osteosclerosis and degeneration of the lateral superior anterior acetabulum was observed, so we added TPO.

After surgery, the surgical side was fixed with a hip spica cast for 6 weeks (Fig. 1b). For 1 year after the surgery, non-weight-bearing walking using a crutch was promoted. Plain X-ray 1 year after the surgery confirmed improvements in joint space narrowing of the left hip and acetabular/femoral head osteosclerosis. Full-weight-bearing walking was then initiated (Fig. 1c).

2 years after the surgery, coxalgia resolved and implant removal was performed (Fig. 1d). The range of motion (lt/rt) was 120/120 degrees on flexion, 30/40 degrees on abduction, 40/40 degrees on internal rotation, 40/40 degrees on external rotation, and 10/10 degrees on adduction, demonstrating improvement. Walking was possible; however, a difference in the leg length was identified, with the affected side lower limb approximately 3 cm shorter than the unaffected side lower limb.

### Case 2

The patient was a 13-year-old girl. She was brought to our hospital with left coxalgia and claudication. She had fallen 2 months previously, and the gluteal region was bruised. However, no abnormality was noted on plain X-ray, and pain disappeared after 1 month. Before this episode, there was no history of coxalgia, claudication, DDH, or generalized joint laxity. Concerning sports, she had played tennis for the previous year.

The range of motion in her hips at the initial consultation (lt/rt) was 90/120 degrees on flexion, 30/40 degrees on abduction, 40/40 degrees on internal rotation, 20/40 degrees on external rotation, and 15/15 degrees of adduction. On abduction and external rotation, the range of motion was limited, and the FADIR test demonstrated negative reactions in bilateral hips.

**Fig. 4:**
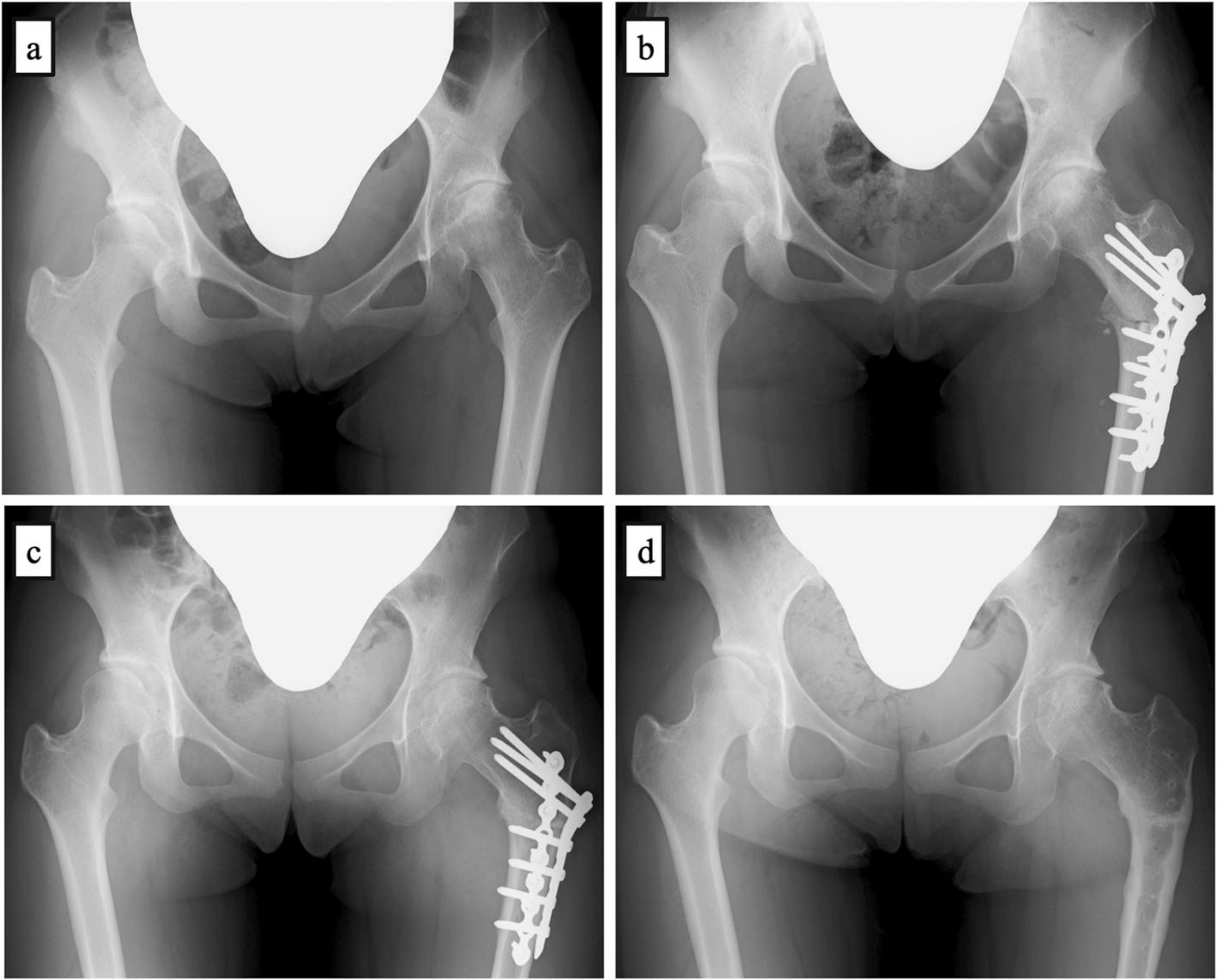
Radiography AP view (a: preoperation, b: postoperation, c: 1 year after surgery, d: 1.5 years after surgery). a: Irregularity of the left anterolateral femoral head and a subcartilaginous cyst are seen. Lateral center-edge, Tonnis, and Sharp’s angles (lt/rt) were 25/25, 10/10, and 45/45 degrees, respectively. There were no signs of femoroacetabular impingement. b: Immediately after surgery. c: 1 year after surgery. Joint space narrowing and femoral head osteosclerosis improved, and there was improved hip conformity. d: 1.5 years after surgery. Implant removal

Plain X-ray revealed the disappearance of the Y cartilage, irregularity of the left anterolateral femoral head, and a subcartilaginous cyst. The LCEA (lt/rt), Tonnis angle, and Sharp’s angle were 25/25, 10/10, and 45/45 degrees, respectively. The head–neck offset ratio was 0.22. And the alpha angle was 39 degrees. There was no disruption of Shenton’s line, or acetabular retroversion, cross-over sign, pistol grip deformity, or herniation pit on either side (Fig. 4a).

**Fig. 5:**
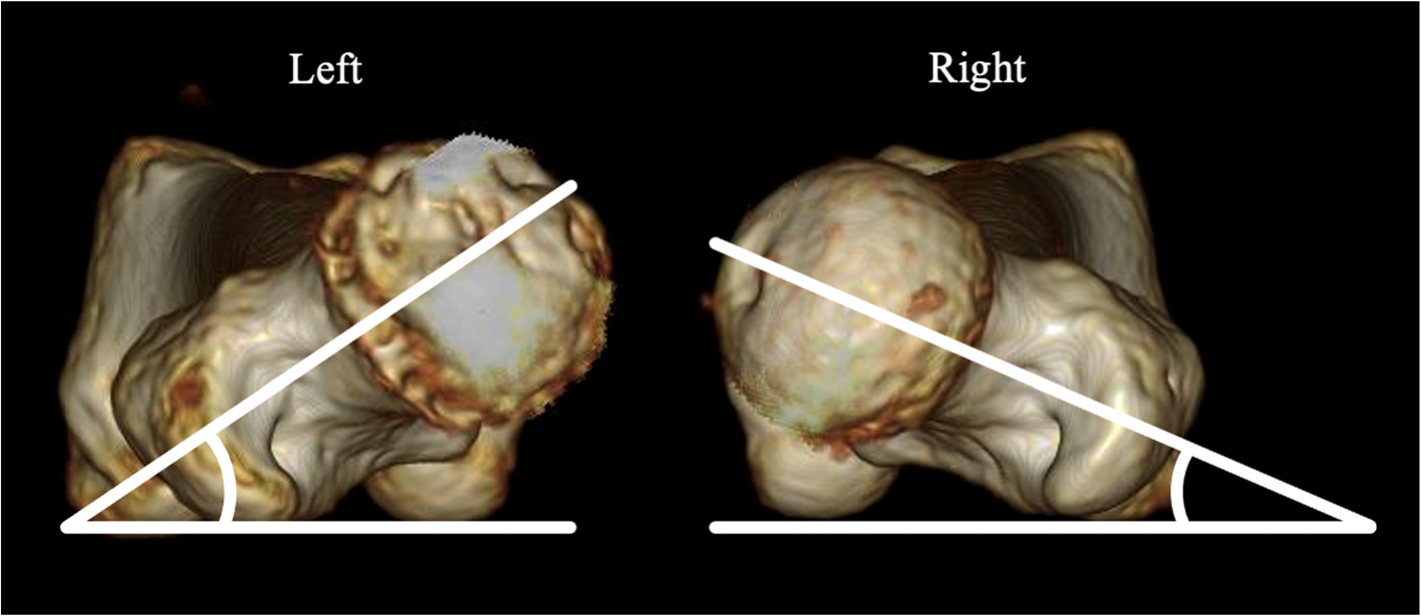
Plain 3D-CT. The anteversion angle of the femoral necks (left/right) was 30/20 degrees. Furthermore, osteophyte formation on the anterior femoral head is present

**Fig. 6:**
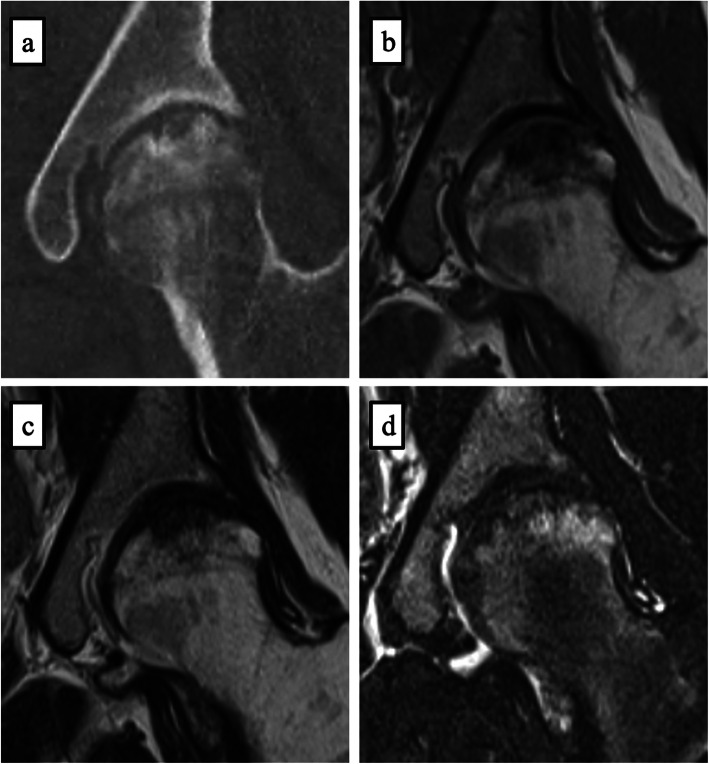
Plane CT and MRI (a: Coronal view of plane CT, b: T1 weighted image, c: T2 weighted image, d: Short TI inversion Recovery). Subcartilaginous cyst in the femoral head and edema were observed

CT revealed irregularity of the left anterolateral femoral head and a subcartilaginous cyst. The anterior center edge angle (ACEA) for the evaluation of acetabular coverage (lt/rt) was 53/58. The femoral neck shaft angle (lt/rt) was 130/130 degrees, and the anteversion angle of the femoral neck (lt/rt) was 30/20 degrees [9] (Fig. 5). Necrosis of the femoral head was not found on MRI, but a subcartilaginous cyst in the femoral head and edema were observed (Fig. 6). There were no abnormalities in the blood test.

A diagnosis of unilateral premature osteoarthritis of the left hip with excessive anteversion of the femoral neck was made. She was prohibited from playing sports and prescribed rest. Pain decreased after 1 month, and there was no limit in the range of motion in the left hip; however, coxalgia recurred 1 year and 6 months after the initial consultation. The range of motion in her hips (lt/rt) was 30/120 degrees on flexion, 10/40 degrees on abduction, 20/40 degrees on internal rotation, 20/40 degrees on external rotation, and 15/15 degrees of adduction. On abduction, internal rotation, and external rotation, the range of motion was limited, and surgery was selected. Plain X-ray demonstrated joint space narrowing and osteosclerosis of the femoral head. As hip conformity on extension/internal rotation/abduction was favorable, PFFDVO (flexion: 20 degrees, detorsion: 20 degrees, inversion: 20 degrees) was performed to improve hip conformity, reduce anteversion of the femoral neck, and alter the weighted surface of the femoral head. In addition, we resected bone spurs on the femoral head because flexion was limited owing to the presence of osteophytes on the anterior femoral head. Acetabular osteotomy was not added because there was little osteosclerosis of the acetabulum, and the patient wanted to be discharged early.

After surgery, non-weight-bearing walking with a crutch was promoted (Fig. 4b). Plain X-ray confirmed improvements in joint space narrowing and osteosclerosis of the femoral head 1 year after surgery, at which time, full-weight-bearing walking was initiated (Fig. 4c).

1.5 years after the surgery, coxalgia resolved and implant removal was performed (Fig. 4d). The range of motion (lt/rt) was 90/120 degrees on flexion, 20/40 degrees on abduction, 20/40 degrees on internal rotation, 20/40 degrees on external rotation, and 15/15 degrees of adduction, demonstrating improvements. Walking became possible; however, range-of-motion limits remained (flexion: 90 degrees, abduction: 20 degrees, internal rotation: 20 degrees, external rotation: 20 degrees).

## Discussion and Conclusions

We described two patients with unilateral OA of the hip early in their teens in whom the anteversion angle of the affected femoral neck was greater than that on the unaffected side, and who had no history of DDH or FAI. The patients underwent PFFDVO + TPO and PFFDVO + bone spur resection on the femoral head, respectively. The two surgical methods led to resolution of the patients’ coxalgia and claudication/gait disorder, although only the short-term results were reviewed. Plain-X-ray confirmed improvements in joint space narrowing and acetabular/femoral head osteosclerosis. However, in the patient treated by PFFDVO + TPO, there was a difference in the leg length. In the other patient, who underwent PFFDVO + bone spur resection of the femoral head, range-of-motion limits remained.

Few studies have reported the onset of OA in the early teens. Only two patients have been reported: one treated by hip arthroscopy by Nishikino et al. [1] and one in whom femoral varus osteotomy was performed by Baba et al. [2]. Both patients were unicycle athletes, suggesting an association of OA with sports-related FAI.

Regarding the association between FAI and OA, Agricola et al. reported that cam-type (alpha angle: ≥ 65 degrees) FAI progressed to terminal OA [11, 12]. Thomas et al. found that the risk of OA progression increased by 5 % with a 1-degree increase in the alpha angle of ≥ 65 degrees, and that the possibility of total hip arthroplasty (THA) indication increased by 4 % with a 1-degree increase [13]. In contrast, Anderson et al. reported that there was no relationship between the alpha angle and OA progression [14]. These studies involved adults, and the incidence of FAI in the general population is unclear; therefore, whether the condition is abnormal remains to be clarified [15], and an association between FAI and OA cannot be concluded.

Vingard et al. reported an association of OA with the type of sports and reported that risk factors for OA included athletics and racket sports [16]. Furthermore, Palmer et al. reported that the incidence of cam-type FAI increased with age in football club players aged 9–18 years [3]. In our Case 2, the patient played tennis, but Vingard et al. investigated middle-aged adults, and Palmer et al. examined football club players; whether OA was associated with sports in our Case 2 remains to be clarified.

Previous studies examined the association between DDH and OA. Patients with an LCEA of < 20 degrees are considered to have dysplasia; those with an LCEA of 20 to < 25 degrees are considered to have borderline dysplasia; and an LCEA of 25 to < 39 degrees is considered a normal range. [5]. Agricola et al. reported that an LCEA of < 25 degrees was a risk factor for OA [12]. Thomas et al. noted that the risk of OA progression increased by 13 % with a 1-degree decrease in LCEA of < 28 degrees, and that the risk of THA increased by 18 % with a 1-degree decrease [13]. Anterior center edge angle (ACEA) for the evaluation of acetabular coverage measured using radiograph false profile view or CT. Nakahara et al. reported that the mean ACEA was 23.1 degrees in DDH and 58.6 degrees in normal hip [17]. In our 2 cases, DDH cannot be completely denied, because LCEA were borderline and ACEA were normal, but there was no significant difference between the affected side and the unaffected side.

Regarding the mechanism of DDH, malposition-related loading of the lateral superior anterior femoral head and a portion of the acetabulum may induce cartilage injury, leading to OA [18]. However, in both of our patients, there was no history or finding of DDH, and plain X-ray images demonstrated normal findings.

As described above, few studies have reported the onset of OA in the early teens, excluding reports in patients with FAI, sports-related injuries, and patients with DDH. In both of our patients, the effect of sports history was unclear, but there was no FAI or DDH. However, the anteversion angle of the femoral neck on the affected side was greater than that on the unaffected side in both patients. Anteversion of the femoral neck is frequently observed in DDH patients, but its involvement in OA remains to be clarified [7].

Douglas et al. reported that anteversion of the femoral neck was not involved in OA in adults [8]. However, the authors did not compare the angle of anteversion on the affected side with that on the unaffected side. Akiyama et al. reported the femoral anteversion had wide variations, and the average femoral anteversion was 22.2 ± 10.8 degrees in dysplastic hips and 14.3 ± 6.8 degrees in normal hips [19]. Nakahara et al. reported the mean femoral anteversion was 38.9 degrees in DDH and 25.1 degrees in normal hip [17] In our two patients, there was no remarkable abnormality except that the anteversion was a little large. There was greater anteversion angle of the femoral neck on the affected side compared with that on the unaffected side; therefore, affected side anteversion of the femoral neck at an angle greater than that on the unaffected side may be involved in the pathogenesis of OA in the early teens. Someone will wonder if the 10 degrees difference has pathological significance. We believe that it is pathologically significant because the anteversion does not change over the age of 8 to 10 and there is no difference between the affected and unaffected sides in most cases.

Few studies have reported treatment for OA in the early teens, and no treatment method has been established. In our patients, the anteversion angle of the femoral neck on the affected side was greater than that on the unaffected side. Furthermore, in Case 1, joint space narrowing and osteosclerosis of the lateral superior anterior acetabulum were observed. In both Cases 1 and 2, osteosclerosis and osteophyte formation on the anterolateral femoral head were marked; therefore, loading of the lateral superior anterior acetabulum related to affected-side anteversion of the femoral neck at an angle greater than that on the unaffected side may have induced acetabular/femoral head cartilage injury. PFFDVO was performed because hip conformity on extension/internal rotation/abduction was favorable. In addition, in Case 1, joint space narrowing and osteosclerosis of the lateral superior anterior acetabulum were marked, and TPO was performed. In Case 2, osteophyte formation on the anterior femoral head was present, and flexion was limited; therefore, bone spur resection on the femoral head was performed. In both patients, coxalgia and claudication/gait disorder resolved postoperatively, and joint space narrowing and osteosclerosis improved, suggesting that affected side anteversion of the femoral neck at an angle greater than that on the unaffected side is involved in the pathogenesis of OA. However, in Case 1, there was a 3-cm difference in the leg length, and in Case 2, range-of-motion limits remained. Over time, our patients’ leg-length difference or range-of-motion limits may lead to recurrent OA; long-term follow-up is necessary.

We described two patients with unilateral OA in their early second decade, with no history of FAI or DDH, in whom the femoral anteversion angle on the affected side was greater than that on the unaffected side. One patient underwent PFFDVO + TPO, and PFFDVO + bone spur resection of the femoral head was performed in the other patient. Coxalgia and claudication/gait disorder resolved postoperatively, and plain X-ray images demonstrated improvements in joint space narrowing and acetabular/femoral head osteosclerosis. However, a difference in the leg length in one patient and range-of-motion limits in the other patient remained.

## Data Availability

Not applicable.

## Abbreviations

OA: Osteoarthritis of the hip
FAI: Femoroacetabular impingement
DDH: Developmental dysplasia of the hip
DDH: Developmental dysplasia of the hip
FADIR: Flexion, adduction, and internal rotation
LCEA: Lateral center-edge angle
CT: Computed tomography
ACEA: Anterior center edge angle
MRI: Magnetic resonance imaging
PFFDVO: Proximal femoral flexional derotation varus osteotomy
TPO: Triple pelvic osteotomy
THA: Total hip arthroplasty
